# Robert Bentley Todd

**Published:** 1861-01

**Authors:** 


### In Memoriam.

### (ROBERT BENTLEY TODD.)

GREAT professional distinction naturally attracts the notice and admiration of all who witness it. The merits, the talents, and the qualifications that have secured remarkable success, must possess personal interest for those aspiring to a similar position. The education, the friendships, the pursuits of every man who achieves pre-eminence, are anxiously investigated by many who seek in them the motives by which his conduct was actuated, and the secret whereby the summit of ambition may be attained.

Professional life, its triumphs and failures, its duties and responsibilities, its joys and sorrows, acquire solemn interest when illustrated by the career of one who has recently left its scene. While the recollections of friendship are fresh, it is difficult to preserve to their scrutiny a strictly impartial judgment. Inasmuch as a future age will be eager in its inquiries concerning men who have exercised an influence on professional councils, and held a high place amongst professional brethren, it is befitting that their cotemporaries should adequately estimate the singular assiduity, indefatigable industry, and rare ability, by the persevering exercise of which any individual has been regarded as able, useful, and eminent.

The last year has been marked by the death of many great and justly-distinguished men, whose names, renowned in the councils, or proved in the defence of their country, are numbered with those whom posterity will delight to honour. Their deeds remain to be chronicled in the history of their time.

It is our sad privilege to investigate a career of equal utility though of less ambition, and to point specially to one on whom distinction was conferred by talent and experience in a profession to whose keeping is entrusted much of the comfort and happiness of social life.

ROBERT BENTLEY TODD, whose writings we have frequently quoted, sometimes in terms of dissent, always with respect, was the second son of Charles Hawkes Todd, Professor of Anatomy and Surgery in the Royal College of Surgeons in Ireland. A marble bust in the great hall of that institution, testifies by a suitable inscription, the esteem in which his father was held. His sudden death at the early age of forty-six years, in the zenith of professional fame, before it had eventuated in the acquisition of fortune, seriously affected the prospects of his youthful and numerous family. Robert Bentley, his second son, was originally designed for the bar, and had, while completing his undergraduate course in Trinity College, kept the terms required in the Dublin Inns. The great calamity of his father's death, while it prevented the further carrying out of his intentions in this respect, at the same time aroused on his behalf the sympathies of numerous friends, who urged his adoption of the medical profession as offering, in his altered family circumstances, greater facilities for the acquisition of an immediate income, and for the obtainment of practice on which he would enter with unusual advantages. Acting on their advice, Mr. Todd abandoned his original intention, and commenced medical and surgical studies with a mind well schooled in classic and scientific learning, and habituated from its previous pursuits to close reasoning and analysis. The kindness of the late Mr. Peile insured him every facility for doing so. The Richmond Hospital, with its collateral institutions, afforded the most extensive opportunities for acquiring a complete professional education. Of these the new student most sedulously availed himself.

At this period a spirit of honourable rivalry characterized the teachers in the various Dublin hospitals. The profound knowledge of Graves and the rising genius of Stokes had inaugurated that system of clinical instruction which has since rendered their schools famous. Each sick-ward became a text-book for the exposition of disease. The impetus of competition among teachers was reciprocated by industry among students. Active pathological research constituted each anatomical theatre an arena wherein the problem of vital symptoms and material changes in their mutual reactions was ultimately investigated, if not solved. Morbid anatomy in its relation to vital pathology—changes after death in their connexion with indications during life—became objects of special study. Mr. Todd was distinguished as the most diligent of pupils. Then, no doubt, he became impressed witht hat spirit of medical eclecticism which subsequently characterized his practical writings, and constituted the chief peculiarity of his teaching. There he was taught that experience, reducing philosophy to practice, finds much in the principle of life inexplicable by the laws of matter: much that reason cannot solve, but which the physician must not therefore hesitate to encounter. As resident pupil in the hospital, Mr. Todd was led to the frequent investigation of disease in its post-mortem indications, and so laid the foundation of that disposition for pathological research which exercised so material an influence on his ubsequent career. After the usual probationary studies he was admitted as a Licentiate of the Royal College of Surgeons in Ireland on the 25th of May, 1831. His father's name and reputation were still cherished in Dublin. Bright prospects seemed to greet the young practitioner. With a view to the further perfecting of his education, he resolved to visit the continental schools. He arrived in London *en route*, when circumstances occasioned a change in his intentions. Shortly after his arrival in the metropolis a vacancy occurred in the Lectureship on Anatomy in the Aldersgate School of Medicine. For this appointment he was induced, by the late Mr. Travers, to become a candidate. His father's high reputation, his own known industry and opportunities secured his election. That internal confidence in his talents which is inseparable from greatness of mind enabled him to sustain its responsibilities. Thus, within a few months of his admission to the profession, we find him actively and perseveringly engaged in lecturing on and demonstrating anatomy, a branch of professional learning which, more than all others, demands unabating industry, close study, and practical acquaintance with its subjects.

Singularly assiduous and indefatigable in the duties of his position, the young lecturer soon established a reputation for accuracy and painstaking research which insured the attention and respect of his class. He resigned this appointment to join the school of the Westminster Hospital, which afforded a wider field for carrying out his favourite researches. At this period, twenty-five years ago, he projected his great undertaking the *Cyclopædia of Anatomy and Physiology*. The extraordinary merits of this work, in connexion with the then knowledge on the subjects treated, at once recommended it to the profession. In the year 1836, Mr. H. J. Rose, Principal of King's College, impressed with the great ability of the work, and the personal character of its author, induced him to seek the Professorship of Physiology and Morbid Anatomy in that College. To this distinguished position he was elected. About the same period he took an *ad eundem* degree in arts in the University of Oxford, and proceeded to the degree of M.D. in Pembroke College. Shortly afterwards he became a Licentiate of the Royal College of Physicians of London, and was appointed physician to the Western Dispensary and the Royal Infirmary for Children. In the following year he was admitted to the fellowship of the College of Physicians, and a few months subsequently elected a Fellow of the Royal Society. Dr. Todd was now in his twenty-ninth year. He had been resident in London but six years. We know of no similar instance of such a rapid success. Arriving among strangers without introduction, wanting those adventitious aids which enable men in professional life to work and wait their time, each year brought him new rewards, the result of self-denying industry and untiring perseverance. At an age when many enter life, he had achieved high professional honours, and taken his stand on equal ground in the most distinguished scientific society of Europe.

Dr. Todd's success was self-acquired, the offspring of his individual merit. The medical youth of England will do well to reflect on the means by which it was accomplished. Habits guided by well-regulated prudence, which at the same time creates and preserves a fortune, maintained in their proper relation his expenditure and income. Each hour of relaxation devoted to literary pursuits, the remainder of his time occupied with professional business, left little opportunity for those temptations with which all large cities abound. That indefatigable spirit and purpose which are characteristics of high intellect; that unabated ardour which amid embarrassments of situation carries its possessor beyond the present, and causes immediate difficulties to be regarded as but further incentives to exertion; that probity of principle which secures friends, that simplicity of manners which disarms enemies, enabled him to conquer and overcome every obstacle, and in a brief time to be recognised as an authority on abstract questions of science, and esteemed as a worthy colleague by men of exalted station among a community into which but a few years before he had ventured to enter, to use his own honest but proud words, "without a sixpence to help him," and with scarcely a single friend.

How many young men commencing the practice of their profession lose sight of the great fact, that opportunity is as frequently the creation of ability as the occasion for its exposition! How many indulge the delusive belief that chance will supply what genius may command or industry accomplish! How many expect to reap rewards similar to those gained by others, yet care not to perform their labour! How many shrink from the patient, self-denying, self-sacrificing devotion to science, without which, success is meretricious, and fame must prove insecure! Let such thoughts be no longer entertained. He is not fit for professional success who does not, at his entrance on professional study, prepare his mind for an ordeal similar to that we have just illustrated; who, content to sacrifice his pleasures on the altar of science, does not find in duty performed a self-sustaining source of satisfaction, which imparts honour by anticipation, and points to merit as the only safe and certain road to independence and fame.

Henceforth we must consider Dr. Todd as occupying a new position. His connexion with King's College contributed in no inconsiderable degree to the foundation of the admirable hospital now bearing that name, within whose walls so many pupils have benefited by his instructions. There can be no doubt that the character of his teaching has exercised a sensible influence on the preliminary education of the medical student. Dr. Todd regarded anatomy and physiology as sciences of the first importance. He esteemed a knowledge of the laws of life, and of its organism in health, to be the only sure basis on which pathology, as a science, can rest. He therefore eagerly adopted the several material aids for physical analysis which progressing science afforded, and found in the microscope and test-tube means for a closer approximation to the material changes perfected by agencies beyond the reach of either. A less enlarged mind would have succumbed to the apparent certainty of materialism, and so have reduced science to routine ; or, finding much that was inexplicable in the operation of disease, would have become sceptical, and lost confidence in the resources of his art. Dr. Todd committed neither of these errors. By a constant regard to the results of actual experience, as well as by the dictates of an enlarged reason: by a fixed determination always to be practical, at the same time giving scope to the most extensive general views : by a cautious and prudent abstinence from every extreme : by investigating, comparing, and pursuing the analogies of things, and, when practicable, tracing events to their remote origin, he rendered science subservient to the purposes of life, and reduced to materials for practice the suggestions of philosophy. As his observation of disease became more extensive, in the formation of his opinions he well and warily propounded his practical deductions, and although he viewed many subjects in different lights at the earlier and later periods of his career, he did so because the soundness of a more mature and practical judgment led to enlargement of speculative views, while a cautious circumspection of surrounding combinations resulted in a provident foresight into possible consequences. A careful regard for vital facts which observation of disease alone can give, as well as a more extended consideration of abstract principles which experience can alone impart, led him, in the diagnosis of affections and the application of remedies, to the nicer weighing of opposite arguments and the acuter perception of practical consequences.

There can be no doubt that a mind such as his, capable of detecting the wants, and a genius formed by nature to supply the deficiencies which observation discovered, would, had Providence so willed it, have accomplished lasting benefits for that science to which he was so entirely devoted. As it is, he has left great results behind him. In an age fruitful with discovery, he must be regarded, if not as the founder, certainly as the chief apostle of, and most active labourer in a school which offers the fascination of atomic analysis. We rejoice to record that he lived to expound its true value.

Of the character of Dr. Todd's clinical teaching it would be impossible to speak too highly. He duly estimated the great importance and duties of a position which constitutes each hospital physician the responsible guide of those youths who are ultimately destined to have the most serious trusts committed to them. He lost no opportunity of impressing on all entering the medical profession the careful cultivation of habits of clinical observation and bed-side study. Looking upon medicine as an art, he regarded accuracy in diagnosis as the essential precursor of all safe treatment: viewing its practice as a science, with enlarged views of life, he carefully watched the operation of remedies. Possessing a quickness of apprehension which enabled him to see at a glance that which cost other minds the labour of an investigation, he yet, in the formation of the student's experience, proceeded to the consideration of circumstances that seemed to be of the most trifling account. So gradually developing to the young mind the necessity of careful research, he smoothed the difficulties in the beginner's progress, and encouraged him to a confidence in his untried capabilities of acquiring intimacy with disease. In this manner he secured the confidence and grateful affection of his class.

While thus perfecting his reputation as a sound practical physician, Dr. Todd by no means abated his labours on abstract science. In the year 1845, he published, in conjunction with Mr. Bowman, his *Physiological Anatomy*, which immediately became the standard work on the subject of which it treats. The *Cyclopædia*, by its recurrent appearance, reminded the profession that the practising physician still continued to labour in his closet. This work, which in its completion occupied the most active period of his professional life, was not brought to a close till a year before his death. This it was which first secured to him reputation as an author, and must ever be regarded as a monument of his industry. Yet inasmuch as a quarter of a century, over which its edition extends, is an age in science, we doubt, if he could have calculated that period, he would have undertaken so herculean a task. There is reason to believe that its publication, if it did not retard, certainly in no commensurate degree promoted his position as a practising physician. The idea got abroad that the cultivation of speculative science was inconsistent with practical observation; but the appearance of his clinical lectures soon removed this erroneous impression, as they at once satisfied the profession and public beyond the sphere of immediate judgment, of their author's capacity for the treatment of disease. In these lectures he manifested a power of analytical and inductive investigation in every department of medical science, which, while enlarging the range of knowledge, increased the active powers of art. A large and increasing practice followed their publication. In 1853, Dr. Todd was compelled, by the pressure of private engagements, to resign the Chair of Physiology, which he had so long and ably filled. Progressing practice necessitated his retirement from the duties of attendant physician to that hospital whose name he has rendered familiar throughout Europe. His farewell address was delivered a few months previously to his death.

Thus far have we traced the almost unexampled career of one who reflected, rather than received, a lustre from science. We forbear to enter on any critical analysis of the author when offering this tribute of our admiration and respect to the man. By his clinical teaching his name will live. He knew its value and illustrated its influence in his own career. The close identity of the views he propounds in his last volume on *Certain Acute Diseases*, with those which during his pupilage prevailed in Dublin, will be evidenced by referring to the lectures of Dr. Graves, delivered at that time. Thirty years ago the value of direct stimulation in general and local lesions was impressed by that great physician who pronounced as his own epitaph, "I fed fevers." It in no wise detracts from Dr. Todd's originality, that he supported, by philosophic deductions and with all the weight of his matured experience, the efforts of a worthy fellow-labourer in the great cause of Truth.

The particular circumstances which attended Dr. Todd's death by hæmatemesis are sadly interesting, if only from the close analogy they present to that of his honoured father. Both died from a similar disease. Both were called away suddenly in the meridian of life, while entering on the full enjoyment of professional prosperity. Both have left the impress of their teaching on the age in which they lived. It is sad for his friends to know that Dr. Todd had received more than one intimation that nature demanded repose. For about three years before his fatal attack, traces of albumen and sugar had been discovered in his secretions. He seems, in the earnest pursuit of professional engagements, to have neglected these warnings, and to have, to his latest moment, indulged in the most active mental exercise at the expense of his nervous system and the exhaustion of his physical strength. It followed that when the attack came, the sentinel was worn and weary at his post. The citadel was lost ere the presence of the enemy was recognised. Then with a faint and passive struggle was surrendered to the hands of Death, one to whom all the honours of a deservedly-won position had opened.

Dr. Todd will not soon be forgotten. He worthily upheld that great reputation his father bequeathed to him, rivalling his distinguished brother, who, in his native city, has likewise reached the summit of scientific eminence. He leaves to a young family, by whom he was beloved, a heritage more precious than wealth, in a high and unblemished reputation—to friends and a profession by whom he is mourned, the blameless integrity of a life devoted to their interests and sacrificed in their cause.

We have here briefly sketched a career on which, with sad remembrances, we would fain invite reflection. To the youth entering on the study of an arduous self-devoting profession, it speaks in terms of comfort and encouragement, and tells that labour shall not go without its reward. It bids the stranger who brings with him energy and ability, welcome to that competition wherein science shows no distinction of rank, and the high road to success is merit. In grave remonstrance it appeals to many who, in devotion to their profession, disregard those warnings which each day's experience affords. It bids such live wisely if they would live long. Fame and Fortune demand for their enjoyment health and vigour; he but half accomplishes his mission who attains one at the expense of the other. The grave and great responsibilities of professional life are too frequently permitted to absorb every consideration of self, until, at length, with his hand upon the prize, the victor falls, and cypress is interwoven with laurel in his crown.

---

